# Prevalence and determinants of multiple chronic conditions (MCC) among young adults in Indian households: an analysis of NFHS-5

**DOI:** 10.1186/s41043-024-00560-0

**Published:** 2024-06-04

**Authors:** Geetanjali Takale, Avantika Handore, Angeline Jeyakumar, Swapnil Godbharle

**Affiliations:** 1 Department of Health Sciences, Savitribai Phule Pune University, Ganeshkhind Road, Pune, Maharashtra India; 2 Food Evolution Research Laboratory (FERL), School of Tourism and Hospitality Management, College of Business and Economics, University of Johannesburg, Johannesburg, South Africa; 3 Department of Nutrition, University of Nevada, Reno, NV USA

**Keywords:** Multiple chronic conditions, Young adults, Households, India

## Abstract

**Background:**

Multiple chronic conditions (MCC) are defined as the presence of two or more chronic conditions, that significantly impact health status, functional capacity, quality of life, and overall healthcare management. Despite the significant evidence on chronic disease burden, the co-existence of MCC within a household in low- and middle-income countries (LMICs) is less studied. This study therefore estimates the prevalence of MCC and its determinants among adults in the Indian households.

**Methods:**

Data used in this study were drawn from the fifth round of the National Family Health Survey (NFHS) conducted in 2019–21. Data sets of men (15–54 years) and women (15–49 years) were used for the study. The total sample size of adults for this analysis was N = 239,848. The outcome variable of this study was multiple chronic conditions (MCC) in adults which included a total of nine chronic conditions (hypertension, diabetes, chronic respiratory diseases, chronic kidney disorders, cancer, thyroid disorders, obesity, and heart diseases, consuming alcohol, chewing tobacco, and smoking) documented in NFHS-5. Descriptive statistics and binary logistic regression analysis were used to quantify the results.

**Results:**

A prevalence of 5.5% of MCC in adults emerged from our study. Logistic regression analysis identified that younger age, males (AOR 0.36 (0.33–0.39)), urban areas (AOR 1.11 (1.02–1.17)) as the place of residence, and participants representing SC (AOR 0.89 (0.81–0.97)), and ST (AOR 1.30 (1.17–1.45)), had a higher risk of MCC irrespective of level of education, type of occupation, marital status, or wealth index, and states from any category of social progress.

**Conclusion:**

A 5% prevalence of MCC specifically obesity, substance use, and hypertension calls for integrated efforts aiming at behavior change, and regulatory efforts to prevent further increase of MCC among young adults in India.

**Supplementary Information:**

The online version contains supplementary material available at 10.1186/s41043-024-00560-0.

## Background

Globally, infections and nutritional disorders have emerged as key determinants of mortality and morbidity. An upsurge in the burden of multiple chronic conditions (MCC) is observed in almost one in three of all adults worldwide [1, 2]. MCC is defined as the presence of two or more chronic diseases, that collectively impact health status, function, or quality of life and that require complex healthcare management, decision-making, or coordination [3]. Non-communicable diseases (NCDs) add to the burden of MCC and the WHO reported that 86% of premature deaths due to NCDs occurred in low and middle-income countries (LMICs) [4]. Cardiovascular disease, cancer, chronic lung diseases, and diabetes have been recognized to increase mortality [5].

The growing burden of MCC is well documented in literature from developed countries where between 16 and 57% of the population have more than one chronic disease. Data from the 2018 National Health Interview Survey (NHIS) estimated, 24.6% (61 million) adults had 1 chronic condition, and 27.2% (68 million) had ≥ 2 chronic conditions [6] in the United States of America (USA). Few other countries have estimated the prevalence of MCC such as Spain (24%), Taiwan (17%), Singapore (16%), and India (23%) [7–10]. However, not all represent population prevalence. The trends varied by geographical region, country’s economy and per capita income, gender, age, number of diseases considered for multimorbidity, or study methodology [11]. In developing countries multimorbidity tends to be more prevalent among the wealthier population, while in developed countries it is higher among the poor. Combinations of MCC varies across nations for example in China [12] hypertension with hearing impairment, and in Africa [13] dyslipidemia with hypertension are a few. Characterizing the patterns is a primary step to plan need-based interventions for specific populations.

Further, the place of residence impacts health and disease. People in urban areas have higher incomes and lead a more sedentary lifestyle than their rural counterparts [14, 15]. Besides, there are differences in types of jobs, education, wealth, social security, and health behavior, all of which are significant determinants of chronic diseases [16]. In developing nations maternal undernutrition that leads to stunted children increases the risk of NCDs and chronic conditions in adulthood [17].

Economic development and social progress are both a cause and consequence of multi-morbidity which is yet to be explored (13,14). Healthcare expenditures greatly increase, sometimes exponentially, with each additional chronic condition with greater specialist physician access, emergency department presentations, and hospital admissions. Individuals with MCC experience deterioration in the quality of life, out-of-pocket expenses, medication adherence, inability to work, and symptom control, which impose a high toll on caregivers [2, 11]. In LMICs, the burden of MCC-associated out-of-pocket expenditure (OOPE) is a rising concern both for the health system and the households [18]. The increasing proportion and a further predicted increase of older and younger adults with MCC who will live to advanced ages are greater public health challenges for policymakers [2].

Despite the significant evidence on chronic disease burden, the co-existence of MCC within a household in LMICs is less studied. Additionally, gaps in knowledge regarding the influence of behavioral or lifestyle factors, such as smoking and alcoholism, on the development of MCC are evident. Existing studies show considerable heterogeneity between MCC and its associated behavioral or lifestyle factors. For LMICs to achieve better health outcomes, it is crucial to gain a better understanding of these knowledge gaps. The lessons from developed countries suggest an increased burden of MCC with increased age. The demography of India is unique characterized by a young population. Studying patterns of MCC will enable characterization to plan specific prevention strategies among the young adults to prevent an increase in burden with increasing age. This study, therefore, estimates the prevalence of MCC and its determinants among young adults in Indian households.

## Methods

### Data source

This study utilized secondary data from the National Family Health Survey (NFHS) 5, a nationally representative cross-sectional survey to estimate the prevalence and determinants of MCC among adults in Indian households. The NFHS 5 (2019–21) was conducted under the stewardship of the Ministry of Health and Family Welfare (MoHFW), Government of India (GOI). It provides essential data on health and family welfare, as well as data on levels of fertility, infant and child mortality, maternal and child health, and other health and family welfare indicators by background characteristics at the national and state levels.

### Sample design

The survey adopted a multistage stratified sampling design to provide various demographic and population health outcome indicators. Each district was stratified into urban and rural areas. Each rural stratum is sub-stratified into smaller substrata considering the village population, and the percentage of scheduled castes and scheduled tribes (SC/ST). The Primary sampling unit (PSU) for urban and rural areas was selected according to the percentage of the SC/ST population. In all, 30,456 PSUs were selected across the country, drawn from 707 districts, 28 states, and 8 union territories of India. For rural areas, a sample of villages (cluster) was selected based on the literacy rate of women aged 6 + years. In urban areas, Census Enumeration Blocks (CEBs) (cluster) were selected based on the percentage of the SC/ST population. In the second stage of selection, 22 households per cluster were chosen with an equal probability of systematic selection of households enlisted during the mapping in the selected PSUs. Overall, 30,456 PSUs were selected across the country from 707 districts, and fieldwork was completed in 30,198 PSUs. This survey provided data on 724,115 women and 101,839 men who were successfully interviewed from 636,699 households.

### Study population

NFHS 5 datasets were accessed from the Demographic and Health Surveys (DHS) website (17). The independent variables of the study included data derived from data sets of young adults defined and categorized as per NFHS as men (15–54 years), and women (15–49 years). The data from the household, household members, and birth datasets were excluded because they did not include variables regarding chronic conditions. The current sample is restricted to a matched sub-set of child data under 5 years of age which is a part of a larger data used for another research objective in the same project. Therefore, the present analysis represents, a sub-sample of N = 239,848 adults (18,086 men and 221,762 women).

### Outcome variable

The outcome variable of this study was multiple chronic conditions in adults (MCC). The outcome of interest was computed using the information on nine self-reported chronic conditions (hypertension, diabetes, chronic respiratory diseases, chronic kidney disorders, cancer, thyroid disorders, obesity, and heart diseases, and substance use disorder (which included consuming alcohol, chewing tobacco, and smoking cigarettes) as documented in NFHS-5. All the chronic conditions were coded into binary categories of No—‘0’ and Yes—‘1’.

### Independent variables

The socio-demographic and economic factors, including age (age groups between, 15–30, and 31–54 years), gender (male and female), place of residence (urban and rural), religion (Hindu, Muslim, Christian, and Other), ethnicity (caste, tribe, no caste/tribe/do not know), marital status (never married, married, widowed or separated or divorced), wealth index (poorest, poor, middle, rich, and richest), education (no education, primary, secondary, higher education), occupation (primary, secondary, tertiary occupation). States were categorized according to the social progress index (SPI) [19, 20].

### Definitions of multiple chronic conditions (MCC)

The lack of a single definition of what constitutes an MCC has resulted in high heterogeneity in the estimates. The definition of MCC is varied as per the number of chronic conditions included [2]. The simplest definition of MCC is the presence of two or more chronic diseases [6, 21, 22]. What constituted a chronic disease also varied across the literature [23]. Some studies define chronic conditions by their specific organ system (e.g., chronic lung disease), whereas others differentiate within organ systems (e.g., COPD and interstitial lung disease) [24]. Several indices have been used to measure the number and severity of chronic diseases. However, the most recognized of these is the Charlson Comorbidities Index and its adaptations, originally established to predict mortality in hospital patients [25].

We, therefore, defined MCC as the presence of two or more chronic conditions (hypertension, diabetes, chronic respiratory diseases, chronic kidney disorders, cancer, thyroid disorders, heart diseases, obesity, and substance abuse) that were documented in NFHS 5.

### Statistical analysis

Cross-tabulations and summary statistics were performed to describe the study population. Simple frequencies, summary measures, tables, and figures were used to present the data. Analyses were based on weighted data to account for the complexity of the survey design. Associations between independent and dependent variables were analysed using chi-square test. Variables that showed significance in the binary logistic regression (Crude Odds Ratio -COR) were subjected to multinomial logistic regression (Adjusted Odds Ratio-AOR) and the analyses were performed to assess the risk factors of MCC among young adults. Associations with *p* =  < 0.05 were considered statistically significant. The adjusted odds ratio (AOR) with a 95% confidence interval was also presented in the results. The analysis was done using the statistical package for the social sciences (SPSS) version 23.0 (International Business Machines Corporation-IBM Corp., Armonk, New York).

### Ethical consideration

The present study utilized a secondary data set from the recent NFHS-5 survey with no identifiable information on the survey participants. The survey received ethical clearance from the Institutions Review Board (IRB) of the International Institute for Population Sciences, India. This dataset is available in the public domain for legitimate research purposes. Since it is a secondary dataset there was no need to get any other institution's ethical permission.

## Results

Table 1 represents the distribution of sociodemographic characteristics of study participants. Reported mean age of participants was 28.49 ± 7.90 years. More than 90% of the participants were females. Of the total female participants, about 70% were in the age group of 15–30 years. The majority of the participants belonged to the Hindu religion (71.7%). A substantial proportion of adults were from scheduled castes (males 71.6% and females 80%). Nearly half of the males (44.8%) were engaged in primary occupations. The majority of males (82.6%) and females (86.5%) were married. More than one-third of the population resided in rural areas (78.5%). The reported educational status of participants showed concentration at the secondary level [males (57%) and females (51.3)]. Nearly one-fourth of the adults belonged to the poorest wealth index [males (26.7%) and females (23.9%)]. Health insurance coverage was higher in males (38.1%) as compared to females (28.4%).

**Table 1:** Sociodemographic characteristics of adults (N = 239,848)

Variables	Categories	Male n (%)	Female n (%)
Age (in years)	15–30	8633 (47.7)	156,165 (70.4)
	31–54	9453 (52.3)	65,597 (29.6)
Religion	Hindu	12,961 (71.7)	164,747 (74.3)
	Muslim	2212 (12.2)	31,310 (14.1)
	Christian	2184 (12.1)	16,468 (7.4)
	Others	729 (4)	9237 (4.1)
Ethnicity	Scheduled Caste	12,957 (71.6)	177,409 (80)
	Scheduled Tribe	3977 (22)	32,468 (14.6)
	Other Backward Class	1152 (6.4)	11,885 (5.4)
Occupation	Primary Occupation	8098 (44.8)	5765 (2.6)
	Secondary Occupation	4865 (26.9)	1573 (0.7)
	Tertiary Occupation	2734 (15.1)	1360 (0.6)
	Not working	1447 (8)	24,557 (11.1)
	Others/Not answered	942 (5.2)	188,507 (85)
Marital status	Never married	3018 (16.7)	23,769 (10.7)
	Married	14,941 (82.6)	191,922 (86.5)
	Widowed/Divorced/Separate	127 (0.7)	6071 (2.7)
Place of residence	Urban	3890 (21.5)	46,429 (20.9)
	Rural	14,196 (78.5)	175,333 (79.1)
Level of education	No education	2594 (14.3)	50,192 (22.6)
	Primary	2601 (14.4)	26,725 (12.1)
	Secondary	10,316 (57)	113,780 (51.3)
	Higher	2575 (14.2)	31,065 (14)
Wealth index	Poorest	4821 (26.7)	53,006 (23.9)
	Poorer	4408 (24.4)	50,947 (23)
	Middle	3794 (21)	44,592 (20.1)
	Richer	3272 (18.1)	40,052 (18.1)
	Richest	1791 (9.9)	33,165 (15)
Covered by health insurance	No	11,197 (61.9)	158,710 (71.6)
	Yes	6889 (38.1)	63,052 (28.4)
Social Progress Index	Very low social progress	3118 (17.2)	39,292 (17.7)
	Low social progress	4009 (22.2)	56,202 (25.3)
	Lower middle social progress	4734 (26.2)	73,805 (33.3)
	Upper-middle social progress	2433 (13.5)	22,796 (10.3)
	High social progress	1856 (10.3)	14,043 (6.3)
	Very high social progress	1936 (10.7)	15,624 (7)

**Fig. 1:**
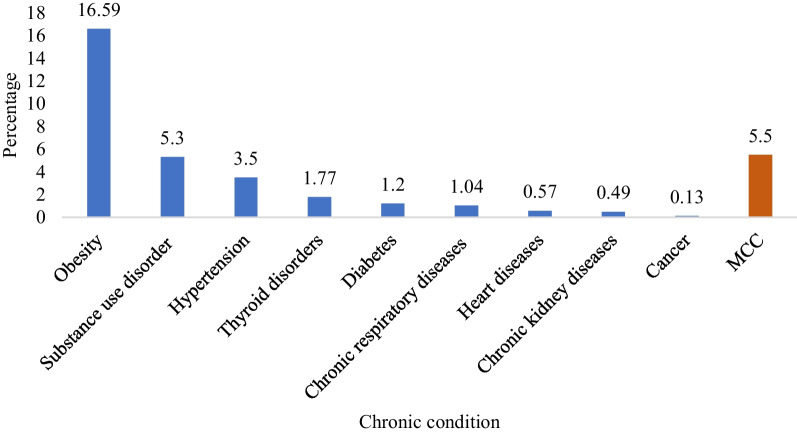
Prevalence of chronic conditions and MCC among adults in India (N = 239,848)

Figure 1 shows the prevalence of nine chronic conditions along with MCC prevalence among adults in India. Among all the chronic conditions, obesity (16.59%) was the most prevalent. Substance use disorder (5.30%) was India's second most prevalent chronic condition, followed by hypertension (3.50%). The prevalence rates of thyroid disorders, diabetes, chronic respiratory diseases, heart diseases, chronic kidney disorders, and cancer were lower than the previously mentioned conditions. However, these chronic conditions are emerging health concerns in India, with prevalence rates ranging from 0.13 to 1.77%. The data shows that the overall burden of MCC in adults was found to be 5.5%.

**Fig. 2:**
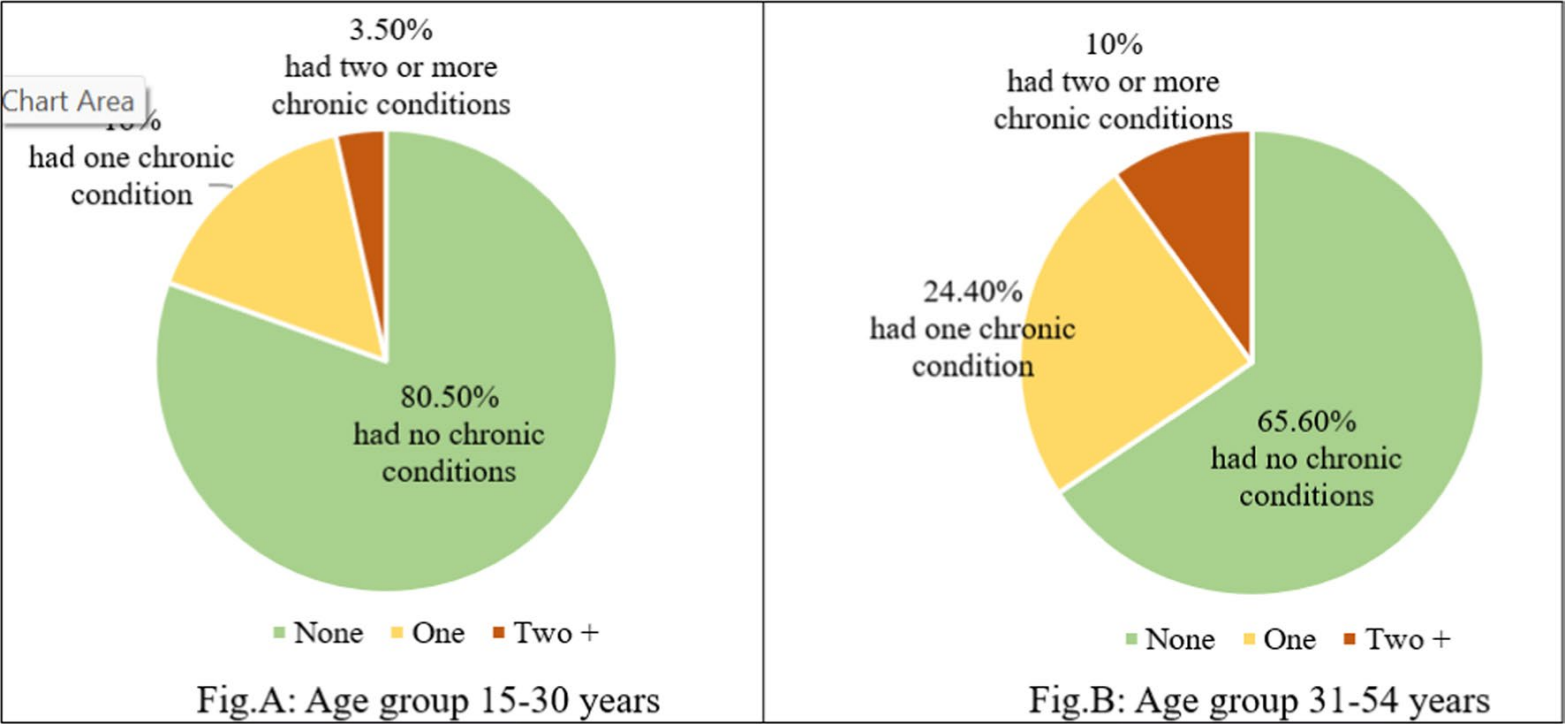
Proportion of adults with and without chronic conditions, by age

Figure 2 shows how chronic conditions are distributed among adults of different age groups. Around 5.5% of all adults had two or more chronic conditions. However, the proportion of people with multiple chronic conditions was almost three times higher in the older age group (31–54 years) compared to the younger age group (15–30 years).

**Fig. 3:**
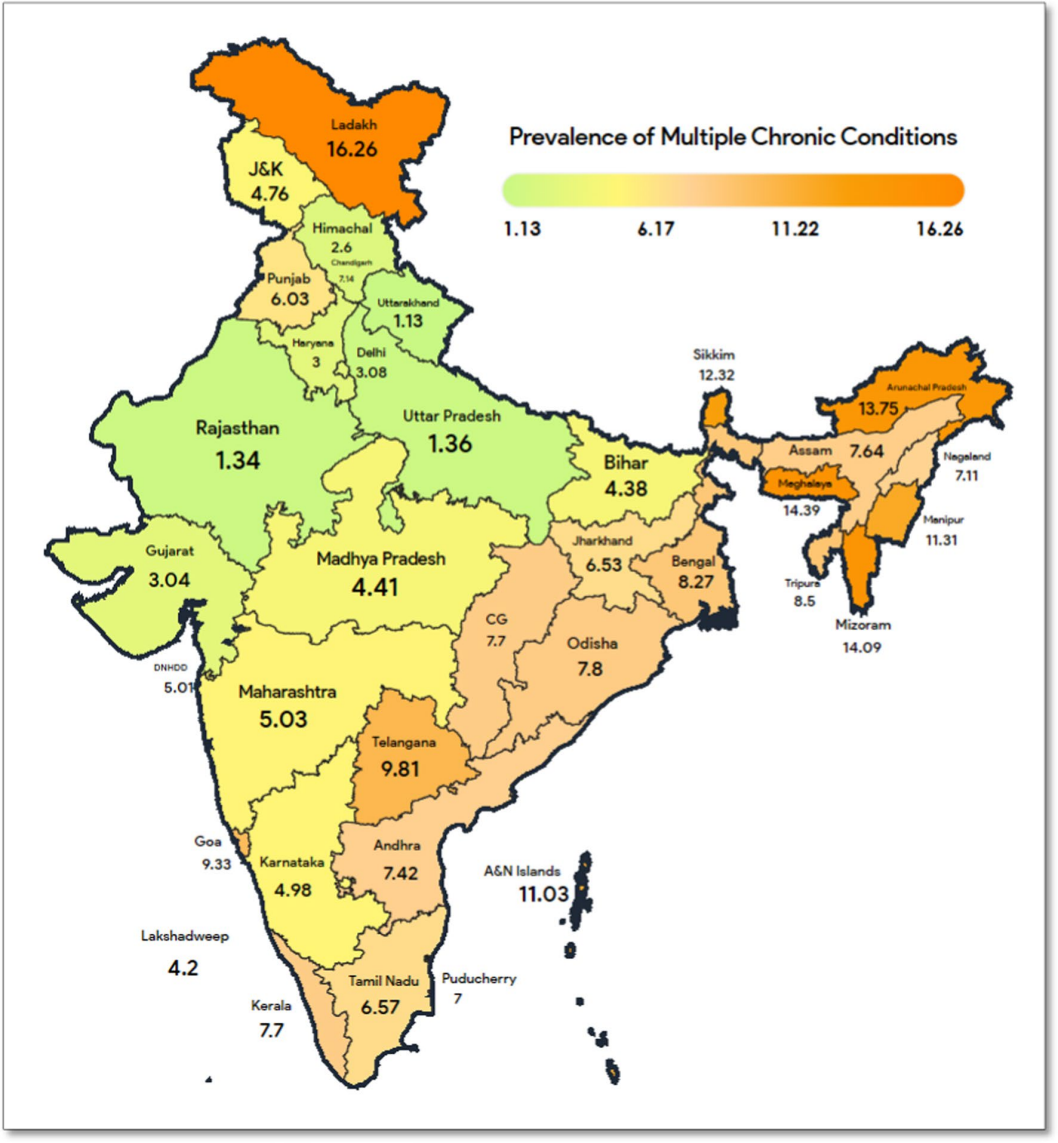
State-wise prevalence of multiple chronic conditions among adults in India (N = 239,848)

Figure 3 presents the state-wise prevalence of MCC among adults in India. The prevalence of MCC was highest in the northern zonal region of India i.e., in Ladakh (16.26%). In the north-eastern region, the highest prevalence of MCC was in Meghalaya (14.39%) and Mizoram (15.09%) followed by Arunachal Pradesh (13.75%). In the eastern region (that consists of states viz. Bihar, Jharkhand, West Bengal, and Odessa) the overall prevalence was between 4.38 and 7.8%. In the central zonal region, the prevalence of MCC was highest in Chhattisgarh at 7.7% and lowest in Uttarakhand at 1.13%. In the western region, the prevalence of MCC was highest in Goa (9.33%) and a similar prevalence was seen in Dadra & Nagar Haveli and Daman & Diu and Maharashtra i.e., 5.01% and 5.03% respectively. In the southern region, a similar pattern of prevalence was seen in Andhra Pradesh, Puducherry, and Kerala i.e., 7.42%, 7%, and 7.7% respectively. But the highest prevalence in this region was in Telangana at 9.81%.

Table 2 shows the percent prevalence of chronic conditions (CCs) in adults according to the social progress index. A high prevalence of obesity (20.8%), thyroid disorders (3.92%), and diabetes (1.65%) was observed in the very high social progress group states while the prevalence of cancer (0.11%) and chronic kidney disorders (0.83%) was low. In the case of the high social progress group, the prevalence of obesity (21%), hypertension (5.59%), and thyroid disorders (3.65%) was higher as compared to other CCs. In the case of upper-middle social progress states, the overall prevalence of chronic conditions was low as compared to other social progress groups. In the lower middle social progress group, the states of Haryana (6.73%), Meghalaya (6.88%), and Tripura (6.02%) showed a higher prevalence of hypertension. Whereas, a greater number of states showed a high prevalence of chronic respiratory diseases i.e., Haryana (0.91%), Gujarat (0.87%), Andhra Pradesh (2%), Meghalaya (3.5%), West Bengal (2.58%) and Tripura (2.23%). The majority of states showed a low prevalence of heart diseases and the overall prevalence was 0.5%. In the low social progress group, a high prevalence of obesity was seen in Odisha (21.3%) and hypertension in Madhya Pradesh (4.55%). Substance use disorders (6.2%) had a high prevalence in the very low social progress group.

**Table 2:**
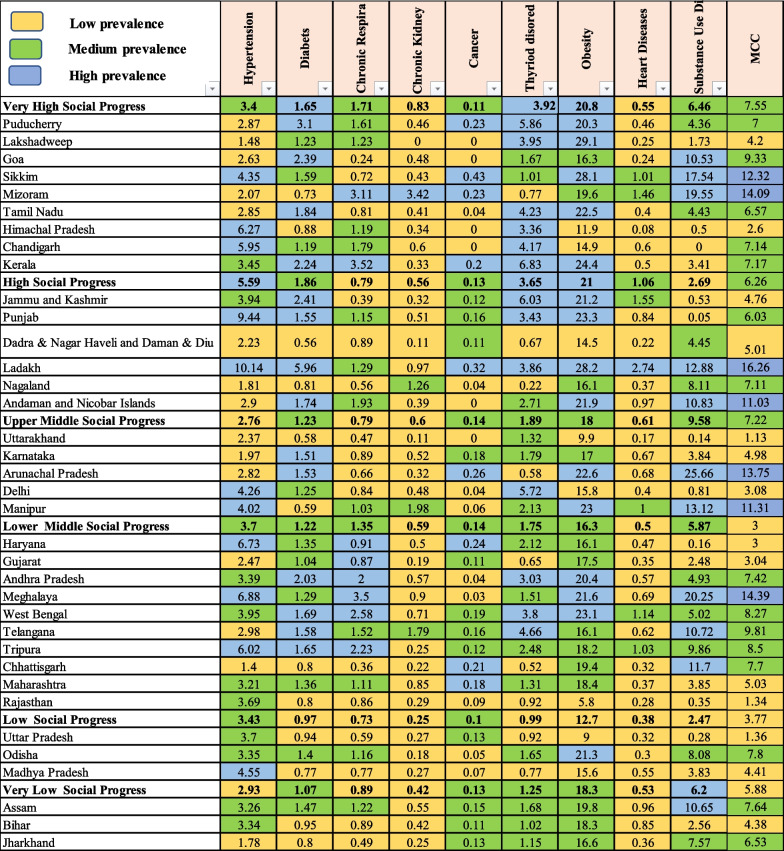
Percent prevalence of chronic conditions in adults according to the social progress index of Indian States/Union Territories

Table 3 shows the logistic regression results of sociodemographic and economic factors associated with MCC among adults. Among the socio-demographic factors considered in the study, the age of the participants 15–30 years [COR 0.569 (0.544–0.595)], being male [COR 0.036 (0.033–0.039)], living in urban areas [COR 1.086 (1.027–0.148)], both scheduled caste and scheduled tribes [COR 0.898 (0.817–0.987)] and [COR 1.306 (1.171–1.455)] respectively, educational status i.e., no education [COR 1.647 (1.514–1.793)], primary [COR 1.674 (1.535–1.826)] and secondary education [COR 1.352 (1.261–1.449)], all levels of occupation including secondary and tertiary occupation [COR 1.390 (1.261–1.531)] and [COR 0.869 (0.768–0.96)] also adults who were not working [COR 0.854 (0.786–0.927)] had higher odds of having multiple chronic conditions. Similarly in the case of marital status both married and never married [COR 0.641 (0.573–0.717)] and [COR 0.382 (0.334–0.438)], adults covered by health insurance [COR 1.175(1.124–1.228)] had higher odds of having MCC (*p* < 0.005).

**Table 3:** Binary logistic regression of the association between sociodemographic characteristics and multiple chronic conditions among adults in India

Variables	Categories	Crude Odds Ratio	95% CI		Adjusted Odds Ratio		95% CI	
		(COR)	Lower	Upper	(A0R)		Lower	Upper
Age (in years)	15–30	0.56***	0.54	0.59	0.56***		0.53	0.59
	31–54							
Sex	Male	0.03***	0.03	0.03	0.36***		0.33	0.39
	Female							
Type of residence	Urban	1.08***	1.02	1.14	1.11***		1.05	1.17
	Rural							
Religion	Hindu	0.74	0.32	1.72	0.72		0.31	1.65
	Muslim	0.59	0.25	1.37	0.57		0.25	1.32
	Christian	1.12	0.48	2.57	1.09		0.47	2.51
	Other							
Ethnicity	Schedule Caste	0.89***	0.81	0.98	0.89**		0.81	0.97
	Schedule Tribe	1.30***	1.17	1.45	1.30***		1.17	1.45
	Other Backward Class							
Education	No education	1.64***	1.51	1.79	1.57***		1.44	1.71
	Primary	1.67***	1.53	1.82	1.61***		1.48	1.76
	Secondary	1.35***	1.26	1.44	1.32***		1.23	1.42
	Higher							
Occupation	Primary	1.06	0.97	1.16	1.06		0.97	1.15
	Secondary	1.39***	1.26	1.53	1.37***		1.25	1.51
	Tertiary	0.85***	0.76	0.96	0.87		0.78	0.98
	Not working	0.85***	0.78	0.92	0.84***		0.78	0.93
	Other / not answered							
Marital Status	Never married	0.38***	0.33	0.43	0.64***		0.57	0.71
	Married	0.64***	0.57	0.71	0.56***		0.53	0.59
	Widowed/Divorced/Separated							
Wealth index	Poorest	0.76***	0.70	0.82	0.82***		0.75	0.89
	Poorer	0.73***	0.68	0.79	0.79***		0.73	0.86
	Middle	0.78***	0.73	0.84	0.82***		0.75	0.88
	Richer	0.81***	0.75	0.87	0.83***		0.77	0.89
	Richest							
Covered by health insurance	Yes	1.17***	1.12	1.22	1.52***		1.47	1.58
	No							
Social Progress Index-State	Very Low Social Progress	0.77***	0.70	0.84	076***		0.71	0.82
	Low Social Progress	0.59***	0.54	0.65	0.40***		0.37	0.43
	Lower Middle Social Progress	0.75***	0.7	0.81	0.76***		0.71	0.81
	Upper Middle Social Progress	0.88***	0.81	0.97	0.95		0.88	1.02
	High Social Progress	1.16***	1.05	1.29	0.81***		0.75	0.89
	Very High Social Progress							

**:<0.01; ***:<0.001

The social progress index complements GDP with quality of life measures such as health, education, infrastructure, etc. When analyzed as per the states social progress index all categories i.e., very low social progress [COR 0.774 (0.709–0.845)], low social progress [COR 0.596 (0.545–0.652)], lower middle social progress [COR 0.758 (0.7–0.819)], upper middle social progress [COR 0.886 (0.81–0.97)] and high social progress [COR 1.169 (1.055–1.297) had higher odds of having MCC (*p* < 0.005).

From all these variables, the younger age group (15–30 years), male adults, urban places of residence, adults representing scheduled caste and scheduled tribes, covered by health insurance, and states with very low social progress stayed significant in the adjusted model as well. In the multinomial regression model, states with low social progress [AOR 0.406 (0.377–0.436)], lower middle social progress [AOR 0.758 (0.715–0.812)], and high social progress [AOR 0.818 (0.751–0.891)] were also statistically significant with MCC.

## Discussion

Our study examined the prevalence and determinants of multiple chronic conditions among adults in Indian households in a nationally representative sample. It is critical to note that 5.50% of adults in the same households have MCC which is a matter of public health concern. The increase in the prevalence of MCC was observed with the advancement of age. Indian traditional family system prioritizes elderly care which increases the need for household resources to manage MCC and meet their health care needs. Among the chronic condition’s obesity, substance use disorder, and hypertension were the most common chronic conditions in India.

### Prevalence of MCC

Our analysis showed obesity, substance use disorder, and hypertension as the primary MCC triad in India. Obesity showed the highest prevalence among chronic conditions. The trend is similar to the earlier rounds of NFHS 2, 3, and 4, which saw an increase in obesity from 8.4 to 15.5% and 2.2 to 5.1% [26–28]. An eight-country analysis of the economic costs of overweight and obesity (OWO) estimated 0.8% of the GDP in India, much lesser compared to the other countries (South Africa, Thailand, Spain, Mexico, Brazil, and Australia) [29]. Luhar and coworkers have predicted a larger relative increase in OWO among the older age groups and in rural compared to urban settings [30]. While the prevalence of cancer was the least in our findings, other countries in the Southeast Asian region showed a high incidence of oral cancer [4]. In the South East Asian region (SEAR) deaths due to chronic diseases ranged from 34% in Timar-Leste to 79% in Maldives [31]. Research from European countries showed smoking as the predominant risk for premature deaths [32]. In the USA percentage population having ≥ 2 chronic conditions was 27.2% much greater than in any other developing country [6]. However, such comparisons need to be carefully interpreted considering the age of the population, the period, the prevailing economic conditions, and other factors like the pandemic that could have contributed to the estimates.

The second highest prevalence in our analysis was substance use disorder (5.3%). In 2019, the Ministry of Social Justice and Empowerment, Government of India reported predominant alcohol use at 4.6% and a dependent pattern use of 19% [33]. The determinants for such high dependency and use of other substances are not limited to biological but also to socio-political factors [34]. The third high prevalent chronic condition in our analysis was hypertension, estimated to be 3.5%. The World Health Organization (WHO) reported hypertension as the major cause of death (27%) among the 40–69 age group [35]. In India, the interventions have strengthened to achieve a 25% reduction in hypertension [36]. Work in India has documented the benefits of a high-protein diet in maintaining normal blood pressure [37].

### Socio demographics and MCC in India

As predicted by Luhar and co-workers (2020), an analysis of socio-demographic characteristics showed MCC was more concentrated in rural than urban areas, where obesity and substance use disorder were prominent [30]. However, our findings reveal a higher prevalence of MCC in urban areas similar to other studies [10, 16]. Among the different ethnic groups, scheduled caste households exhibited a higher prevalence of MCC compared to scheduled tribes. Individuals irrespective of education and occupation were susceptible to MCC. Remarkably, our findings diverged from a previous study, as we did not observe a higher MCC prevalence among unmarried, divorced/separated, or widowed individuals [38].

### Differences in prevalence across states

A high prevalence of chronic conditions was seen among the North and North Eastern (NE) states, Assocham report identified similar patterns in the NE states. These states are in the lower middle stage of epidemiological transition. Their rural and tribal regions have a mountainous terrain that impedes access to health care, characterized by poor infrastructure and increased untreated morbidities. This combined with poverty, high intake of non-vegetarian foods low consumption of fruits and vegetables, and less physical activity adds to the increasing burden of MCC in these states [39, 40]. Our analysis showed a high prevalence of MCC in Chhattisgarh. The high prevalence of NCD, mental health disorders, and substance use above the national average in this state contribute to the high MCC [16, 41]. In our analysis, states in the southern region showed a higher prevalence of MCC compared to the western states. However, such differences have not been observed in previously published work that compared chronic conditions in older adults [42]. A high prevalence is likely to be observed among younger adults that need to be supported by more research.

### Social progress of states and MCC

In our work, states with very high and lower middle social progress displayed higher MCC prevalence in adults. This varied from developed countries where very high economic strata showed a low prevalence of MCC. However, our work used social progress that considered the social context of progress that included education, social conditions that influenced the risk of exposure, environment, degree of susceptibility, the course and outcome of the disease, and not just economic growth [43–45]. In developed countries, the interstate variability was marked, where access to care and insurance varied across degrees of progress [46]. Notably, our study too highlighted that those individuals with health insurance had a lower prevalence of MCC. The findings urge the need to test innovative models for the prevention and control of MCC in developing country settings.

### Strengths and limitations

Our analysis focuses on a less explored area of MCC among young adults in India, with a unique demography, experiencing significant nutrition transition. The strength of our study is the large dataset, covering the nationally representative sample and chronic conditions in young adults as outcomes that had a higher analytical potential. The use of weighted data in our analysis minimized the effect of inherent bias of big datasets especially improving the representation of smaller demographic groups. Many studies focus on individual adults as the study unit, whereas we focused on households relevant to MCC studies. Despite these strengths, our work is not free from constraints. Since these data were derived from a nationally representative cross-sectional survey i.e., NFHS round 5, our findings present the association between the variables but not causation. Further, we used self-reported information on nine chronic conditions, which could not eliminate bias and our study was limited to a small number of conditions. Also, the economic status of the households in this study was measured through the wealth index based on household assets. Additionally, the adult–child matched sub-set within the households’ limits representativeness of young adults. The higher proportion of women in the dataset also could have skewed the study findings. Comparing the prevalence of MCC across different studies poses challenges due to variations in the definition of MCC, data collection methods, and the inclusion of specific chronic conditions.

## Conclusion

The 5% prevalence of MCC among young adults in Indian households represents the tip of the iceberg, that could disguise the huge burden of MCC among the older age groups. Among the major prevalent conditions, obesity, substance use, and hypertension emerge as three major chronic conditions. However, state-wise combined burden identified obesity, combined with thyroid disorders and diabetes as the MCC triad in Indian households. This calls for innovative strategies that collectively address biological and social determinants appropriate for a developing country setting. With the expanding urban population in developing countries, our findings urge concentrated efforts to curb the rising prevalence in urban settings that are well influenced by transition. Adults in mid-life (31–59 years) need priority attention for a country with a young demography, to prevent economic losses. The three major chronic conditions with modifiable determinants and calls to advocate lifestyle modification irrespective of social class and age. In addition, strengthening of health systems, and improving coverage of services to households across settings. The overall findings of the study points to critical and urgent investment in improving the life of young adults and to prevent an increase in the burden of MCC with advancing age. The alarming patterns of MCC seen in developed countries gives India vital lessons to curb progress by investing in the health of young adults. Perhaps integrated efforts aiming at behavior change, alongside regulations for the food industry to promote healthy food environments will be a worthwhile investment.

### Supplementary Information

Supplementary Material 1

## Data Availability

The datasets used and/or analysed during the current study are available from the corresponding author on reasonable request.

## Abbreviations

MCC: Multiple chronic conditions
CC: Chronic condition
NCD: Non-communicable disease
LMICs: Low and middle-income countries
NHIS: National Health Interview Survey
USA: United States of America
OOPE: Out-of-pocket expenditure
NFHS: National Family Health Survey
MoHFW: Ministry of Health and Family Welfare
GOI: Government of India
SC: Scheduled caste
ST: Scheduled tribe
PSU: Primary sampling unit
CEBs: Census Enumeration Blocks
DHS: Demographic and Health Surveys
COR: Crude odds ratio
AOR: Adjusted odds ratio
SPSS: Statistical package for the social sciences
IRB: Institutions Review Board
OWO: Overweight and obesity
SEAR: South East Asian region
WHO: World Health Organization

## References

[1] Sultana M, Mahumud RA, Sarker AR (2017). Burden of chronic illness and associated disabilities in Bangladesh: evidence from the household income and expenditure survey. Chronic Dis Transl Med.

[2] Hajat C, Stein E (2018). The global burden of multiple chronic conditions: a narrative review. Prev Med Rep.

[3] Marengoni A, Angleman S, Melis R, Mangialasche F, Karp A, Garmen A (2011). Aging with multimorbidity: a systematic review of the literature. Ageing Res Rev.

[4] World Health Organization. Non communicable diseases [Internet]. 2023 [cited 2023 Mar 2]. Available from: https://www.who.int/news-room/fact-sheets/detail/noncommunicable-diseases

[5] Wang Z, Locantore N, Haldar K, Ramsheh MY, Beech AS, Ma W (2023). Inflammatory endotype–associated airway microbiome in chronic obstructive pulmonary disease clinical stability and exacerbations: a multicohort longitudinal analysis. Am J Respir Crit Care Med.

[6] Boersma P, Black LI, Ward BW (2020). Prevalence of multiple chronic conditions among US adults, 2018. Prev Chronic Dis.

[7] García-Olmos L, Salvador CH, Alberquilla Á, Lora D, Carmona M, García-Sagredo P, et al. Comorbidity patterns in patients with chronic diseases in general practice. Gagnier JJ, editor. PLoS ONE. 2012;7:e32141. 10.1371/journal.pone.0032141 10.1371/journal.pone.0032141 PMC3281110 22359665

[8] Fu S, Huang N, Chou Y-J (2014). Trends in the prevalence of multiple chronic conditions in Taiwan from 2000 to 2010: a population-based study. Prev Chronic Dis.

[9] Subramaniam M, Abdin E, Picco L, Vaingankar JA, Chong SA (2014). Multiple chronic medical conditions: prevalence and risk factors—results from the Singapore Mental Health Study. Gen Hosp Psychiatry.

[10] Joshi R, Santoshi J, Rai N, Pakhare A (2015). Prevalence and patterns of coexistence of multiple chronic conditions: a study from Indian urban outpatient setting. J Fam Med Prim Care.

[11] Chowdhury SR, Chandra Das D, Sunna TC, Beyene J, Hossain A (2023). Global and regional prevalence of multimorbidity in the adult population in community settings: a systematic review and meta-analysis. eClinicalMedicine.

[12] Hu Y, Wang Z, He H, Pan L, Tu J, Shan G (2024). Prevalence and patterns of multimorbidity in China during 2002–2022: a systematic review and meta-analysis. Ageing Res Rev.

[13] Micklesfield LK, Munthali R, Agongo G, Asiki G, Boua P, Choma SS (2023). Identifying the prevalence and correlates of multimorbidity in middle-aged men and women: a cross-sectional population-based study in four African countries. BMJ Open.

[14] Kim JY, Lee MK, Lee DH, Kang DW, Min JH, Lee JW (2019). Effects of a 12-week home-based exercise program on quality of life, psychological health, and the level of physical activity in colorectal cancer survivors: a randomized controlled trial. Support Care Cancer.

[15] Tripathy JP, Thakur JS, Jeet G, Chawla S, Jain S, Prasad R (2016). Urban rural differences in diet, physical activity and obesity in India: are we witnessing the great Indian equalisation? Results from a cross-sectional STEPS survey. BMC Public Health.

[16] Jana A, Chattopadhyay A. Prevalence and potential determinants of chronic disease among elderly in India: rural-urban perspectives. Mahapatra B, editor. PLOS ONE. 2022;17:e0264937. 10.1371/journal.pone.0264937 10.1371/journal.pone.0264937 PMC8916671 35275937

[17] Black RE, Victora CG, Walker SP, Bhutta ZA, Christian P, De Onis M (2013). Maternal and child undernutrition and overweight in low-income and middle-income countries. The Lancet.

[18] Pati S, Agrawal S, Swain S, Lee JT, Vellakkal S, Hussain MA (2014). Non communicable disease multimorbidity and associated health care utilization and expenditures in India: cross-sectional study. BMC Health Serv Res.

[19] Government of India (GOI). Social Progress Index (SPI) for States and Districts [Internet]. Delhi: Government of India; 2022. Report No.: 1885039. Available from: https://pib.gov.in/pib.gov.in/Pressreleaseshare.aspx?PRID=1885039

[20] International Institute for Population Sciences (IIPS). National Family Health Survey (NFHS - 5), 2019–21 India Report [Internet]. Mumbai: IIPS; 2022 Mar. Available from: https://dhsprogram.com/pubs/pdf/FR375/FR375.pdf

[21] Mira R, Newton T, Sabbah W. Inequalities in the progress of multiple chronic conditions: a systematic review of longitudinal studies. Angkurawaranon C, editor. PLOS ONE. 2022;17:e0263357. 10.1371/journal.pone.0263357 10.1371/journal.pone.0263357 PMC8812855 35113920

[22] Ko D, Bratzke LC, Roberts T (2018). Self-management assessment in multiple chronic conditions: a narrative review of literature. Int J Nurs Stud.

[23] Lefèvre T, d’Ivernois J-F, De Andrade V, Crozet C, Lombrail P, Gagnayre R (2014). What do we mean by multimorbidity? An analysis of the literature on multimorbidity measures, associated factors, and impact on health services organization. Rev DÉpidémiologie Santé Publique.

[24] Diederichs C, Berger K, Bartels DB (2011). The measurement of multiple chronic diseases–a systematic review on existing multimorbidity indices. J Gerontol A Biol Sci Med Sci.

[25] Yurkovich M, Avina-Zubieta JA, Thomas J, Gorenchtein M, Lacaille D (2015). A systematic review identifies valid comorbidity indices derived from administrative health data. J Clin Epidemiol.

[26] National Family Health Survey (NFHS-2), 1998–99 [Internet]. International Institute for Population Sciences (IIPS) and ORC Macro. 2000; Available from: https://www.dhsprogram.com/pubs/pdf/FRIND2/FRIND2.pdf

[27] National Family Health Survey (NFHS-3), 2005–06 [Internet]. International Institute for Population Sciences (IIPS) and Macro International. 2007; Available from: https://dhsprogram.com/pubs/pdf/frind3/frind3-vol1andvol2.pdf

[28] National Family Health Survey (NFHS-4), 2015–16: India [Internet]. : International Institute for Population Sciences (IIPS) and ICF. 2017; Available from: http://rchiips.org/nfhs/nfhs-4Reports/India.pdf

[29] Okunogbe A, Nugent R, Spencer G, Ralston J, Wilding J (2021). Economic impacts of overweight and obesity: current and future estimates for eight countries. BMJ Glob Health.

[30] Luhar S, Timæus IM, Jones R, Cunningham S, Patel SA, Kinra S, et al. Forecasting the prevalence of overweight and obesity in India to 2040. Joe W, editor. PLOS ONE. 2020;15:e0229438. 10.1371/journal.pone.0229438 10.1371/journal.pone.0229438 PMC7039458 32092114

[31] Sharma J (2013). Chronic disease management in the South-East Asia Region: a need to do more. WHO South-East Asia J Public Health.

[32] Muller DC, Murphy N, Johansson M, Ferrari P, Tsilidis KK, Boutron-Ruault M-C (2016). Modifiable causes of premature death in middle-age in Western Europe: results from the EPIC cohort study. BMC Med.

[33] Ambekar A, Agrawal A, Chadda RK, Mishra AK, Rao R, Khandelwal SK. Magnitude of substance use in India. New Delhi: Ministry of Social Justice and Empowerment, Government of India; 2019.

[34] Singh O (2020). Substance use in India—policy implications. Indian J Psychiatry.

[35] World Health Organization. Cardiovascular diseases [Internet]. 2018 [cited 2023 Aug 2]. Available from: https://www.who.int/india/health-topics/cardiovascular-diseases

[36] World Health Organization. India Hypertension Control Initiative, a high impact and low-cost solution [Internet]. 2022 [cited 2023 Aug 2]. Available from: https://www.who.int/india/news/detail/02-06-2022-india-hypertension-control-initiative--a-high-impact-and-low-cost-solution

[37] Gulati S, Misra A, Tiwari R, Sharma M, Pandey RM, Yadav CP (2017). Effect of high-protein meal replacement on weight and cardiometabolic profile in overweight/obese Asian Indians in North India. Br J Nutr.

[38] Rohini C, Jeemon P (2020). Prevalence and patterns of multi-morbidity in the productive age group of 30–69 years: a cross-sectional study in Pathanamthitta District, Kerala. Wellcome Open Res.

[39] Swargiary M, Lhungdim H (2021). Disease burden and healthcare utilization in the north eastern region of India. Demogr India.

[40] Dutta K, Srivastava N. Non-communicable diseases in India [Internet]. India: TARI-ASSOCHAM; 2021. p. 72. Available from: https://tari.co.in/publications/reports/

[41] Singh SK, Chauhan K, Puri P (2023). Chronic non-communicable disease burden among reproductive-age women in India: evidence from recent demographic and health survey. BMC Womens Health.

[42] Patel P, Muhammad T, Sahoo H (2023). The burden of disease-specific multimorbidity among older adults in India and its states: evidence from LASI. BMC Geriatr.

[43] Yang YC, McClintock MK, Kozloski M, Li T (2013). Social isolation and adult mortality: the role of chronic inflammation and sex differences. J Health Soc Behav.

[44] Frohlich KL, Abel T (2014). Environmental justice and health practices: understanding how health inequities arise at the local level. Sociol Health Illn.

[45] Cockerham WC, Hamby BW, Oates GR (2017). The social determinants of chronic disease. Am J Prev Med.

[46] John TJ, Dandona L, Sharma VP, Kakkar M (2011). Continuing challenge of infectious diseases in India. The Lancet.

