# Supplementary material for: Prevalence and determinants of multiple chronic conditions (MCC) among young adults in Indian households: an analysis of NFHS-5
Source: J Health Popul Nutr. 2024 Jun 4;43:77. doi: 10.1186/s41043-024-00560-0 (PMC11149311; doi:10.1186/s41043-024-00560-0)
Supplement: Supplementary file 1 — Supplementary Material 1 [file 41043_2024_560_MOESM1_ESM.docx]

Table: Association between MCC and socio-demographic characteristics of adults

| Variables | Levels | Multiple Chronic Conditions | | Chi-square value |
| --- | --- | --- | --- | --- |
|  |  | Yes n (%) | No n (%) |  |
| Age | 15 - 30 | 5713 (2.38) | 159085 (66.32) | 4158.8* |
|  | 31 - 54 | 7457 (3.10) | 67593 (28.18) |  |
| Sex | Male | 7807 (3.25) | 10279 (4.28) | 53502.6* |
|  | Female | 5353 (2.23) | 216399 (90.22) |  |
| Type of residence | Urban | 2940 (1.22) | 47379 (19.75) | 15.18* |
|  | Rural | 10230 (4.26) | 179299 (74.75) |  |
| Religion | Hindu | 8657 (3.60) | 169051 (70.48) | 1888.8* |
|  | Muslim | 1391 (0.57) | 32131 (13.39) |  |
|  | Christian | 2120 (0.88) | 16532 (6.89) |  |
|  | Other | 1002 (0.41) | 8964 (3.73) |  |
| Ethnicity | Caste | 8826 (3.67) | 181540 (75.68) | 1600.1* |
|  | Tribe | 3587 (1.49) | 32858 (13.69) |  |
|  | No Caste/Tribe/Don’t know | 757 (0.31) | 12280 (5.11) |  |
| Education | No education | 2767 (1.15) | 50019 (20.85) |  |
|  | Primary | 2054 (0.85) | 27272 (11.37) | 203.2* |
|  | Secondary | 6845 (2.85) | 117251 (48.88) |  |
|  | Higher | 1504 (0.62) | 32136 (13.39) |  |
| Occupation | Primary | 3770 (1.57) | 10093 (4.20) | 31798.3* |
|  | Secondary | 2400 (1) | 4038 (1.68) |  |
|  | Tertiary | 1089 (0.45) | 3005 (1.25) |  |
|  | Not working | 902 (0.37) | 25102 (10.46) |  |
|  | Other / not answered | 5009 (2.08) | 184440 (76.89) |  |
| Marital Status | Never married | 1080 (0.45) | 25707 (10.71) | 130.8* |
|  | Married | 11691 (4.87) | 195172 (81.37) |  |
|  | Widowed /Divorced / Separated | 399 (0.16) | 5799 (2.41) |  |
| Wealth index | Poorest | 3506 (1.46) | 54321 (22.64) | 69.4* |
|  | Poorer | 3046 (1.26) | 52309 (21.08) |  |
|  | Middle | 2642 (1.1) | 45744 (19.07) |  |
|  | Richer | 2286 (0.95) | 41038 (17.11) |  |
|  | Richest | 1690 (0.70) | 33266 (13.86) |  |
| Covered by health insurance | No | 8169 (3.40) | 161738 (67.43) | 523.8* |
|  | Yes | 5001 (2.08) | 64940 (27.07) |  |
| States | Very low social progress | 2494 (1.03) | 39916 (16.64) | 946.189* |
|  | Low social progress | 1931 (0.8) | 58280 (24.29) |  |
|  | Lower middle social progress | 4600 (1.91) | 73939 (30.82) |  |
|  | Upper-middle social progress | 1823 (0.76) | 23406 (9.75) |  |
|  | High social progress | 996 (0.41) | 14903 (6.21) |  |
|  | Very high social progress | 1326 (0.55) | 16234 (6.76) |  |

*P<0.05
